# Performance of the New Aptima HCV Quant Dx Assay in Comparison to the Cobas TaqMan HCV2 Test for Use with the High Pure System in Detection and Quantification of Hepatitis C Virus RNA in Plasma or Serum

**DOI:** 10.1128/JCM.03236-15

**Published:** 2016-03-25

**Authors:** Gunnar Schalasta, Andrea Speicher, Anna Börner, Martin Enders

**Affiliations:** Prof. Gisela Enders & Kollegen MVZ GbR, Stuttgart, Germany

## Abstract

Quantitating the level of hepatitis C virus (HCV) RNA is the standard of care for monitoring HCV-infected patients during treatment. The performances of commercially available assays differ for precision, limit of detection, and limit of quantitation (LOQ). Here, we compare the performance of the Hologic Aptima HCV Quant Dx assay (Aptima) to that of the Roche Cobas TaqMan HCV test, version 2.0, using the High Pure system (HPS/CTM), considered a reference assay since it has been used in trials defining clinical decision points in patient care. The assays' performance characteristics were assessed using HCV RNA reference panels and plasma/serum from chronically HCV-infected patients. The agreement between the assays for the 3 reference panels was good, with a difference in quantitation values of <0.5 log. High concordance was demonstrated between the assays for 245 clinical samples (kappa = 0.80; 95% confidence interval [CI], 0.720 to 0.881); however, Aptima detected and/or quantitated 20 samples that HPS/CTM did not detect, while Aptima did not detect 1 sample that was quantitated by HPS/CTM. For the 165 samples quantitated by both assays, the values were highly correlated (*R* = 0.98; *P* < 0.0001). The linearity of quantitation from concentrations of 1.4 to 6 log was excellent for both assays for all HCV genotypes (GT) tested (GT 1a, 1b, 2b, and 3a) (*R*^2^ > 0.99). The assays had similar levels of total and intra-assay variability across all genotypes at concentrations from 1,000 to 25 IU/ml. Aptima had a greater analytical sensitivity, quantitating more than 50% of replicates at 25-IU/ml target. Aptima showed performance characteristics comparable to those of HPS/CTM and increased sensitivity, making it suitable for use as a clinical diagnostic tool on the fully automated Panther platform.

## INTRODUCTION

The number of persons chronically infected with the hepatitis C virus (HCV) is estimated to be 130 to 150 million worldwide (1), including 15 million in the European region (2), making HCV infection an important health concern worldwide. HCV infection is a major cause of chronic liver disease. Although most chronically infected individuals are asymptomatic, 20% to 30% develop cirrhosis over a period of 20 to 30 years, and 1% to 4% of patients with cirrhosis develop hepatocellular carcinoma (HCC) each year (3). End-stage cirrhosis is the leading cause of liver transplantation.

Recently developed therapeutics for the treatment of HCV infection that act directly on the machinery of HCV replication have revolutionized patient care. Combinations of these direct-acting antiviral agents (DAAs) are effective for most patients across all genotypes (GT) with minimal side effects compared to those of traditional therapies, such as pegylated interferon (pIFN) and ribavirin (4–7). The discovery and routine use of ribavarin-free single-tablet formulations continue to advance patient management and may lead to the eradication of HCV infection in the future. The goal of HCV therapy is to cure the infection as judged by a sustained virological response (SVR), which is defined as an undetectable HCV RNA plasma/serum concentration at 12 or 24 weeks after treatment completion using a sensitive HCV RNA quantitation assay with a limit of quantitation of under 25 IU/ml (5). Achievement of SVR is associated with resolution of liver disease in patients without cirrhosis and may lead to regression of fibrosis in those with cirrhosis (5). Furthermore, recent data suggest that the risks of HCC and mortality are reduced in patients achieving SVR compared to the risks in untreated patients (8).

The gold standard for monitoring SVR in HCV-infected patients under treatment is to quantitate HCV RNA in the patient's plasma or serum at regular intervals during and after therapy. Depending on the regimen and the inclusion of IFN, this can include measurement at baseline, week 2 (IFN free), week 4, week 12, week 24, and week 48 (nonresponders for certain regimens) of treatment and 12 or 24 weeks after the end of therapy (5). Not only is HCV RNA monitoring important for determining the initial viral load, it is necessary to assess patient adherence and for following stopping rules to determine when treatment is ineffective and should be discontinued (9–14). This is a necessity for managing side effect profiles, minimizing the emergence of viral resistance, and decreasing the cost of therapy (13, 15). European guidelines recommend stopping treatment with the triple combination pIFN plus ribavirin plus simeprevir if HCV RNA levels are ≥25 IU/ml at week 4, 12, or 24; no futility rules have been defined for other treatment regimens (5). United States guidelines, however, do not provide specific recommendations regarding when to stop or extend therapy except to discontinue treatment if HCV RNA is detectable at week 4 and increases by >10-fold on repeat testing at week 6 (or thereafter) (4). Because these rules are based on a threshold of quantitation of HCV RNA of 25 IU/ml, the guidelines recommend that monitoring be performed using a sensitive real-time quantitative HCV RNA assay, i.e., an assay with a lower limit of quantification (LLOQ) for HCV RNA of ≤25 IU/ml and a limit of detection (LOD) of ≤15 IU/ml (5).

There are currently various commercially available assays for the quantitation of HCV in plasma or serum, all with different performance characteristics (Table 1) (13). Most of these assays use PCR to amplify the HCV 5′ untranslated region (5′-UTR) (the most conserved region of HCV) in plasma or serum samples. The Roche Cobas TaqMan hepatitis C virus test, version 2.0, for use with the High Pure system (HPS/CTM; Roche Molecular Diagnostics, Pleasanton, CA, USA) is considered the reference assay because it was used in phase III clinical trials for approved DAA treatments that included ledipasvir, simeprevir, sofosbuvir, boceprevir, and telaprevir, among others (16–20). The most recently developed HCV RNA quantitation assay is the Aptima HCV Quant Dx (Aptima; Hologic, Inc., San Diego, CA, USA) for monitoring and diagnosis (CE-approved as an *in vitro* diagnostic device [IVD] in Europe), which is used on the fully automated Panther platform. Aptima uses real-time transcription-mediated amplification (TMA) (21) to amplify the RNA target (5′-UTR) of HCV genotypes 1 to 6 (22).

**TABLE 1:** Commercially available assays for quantitation of HCV RNA in patients' plasma or serum samples

Parameter	Value (IU/ml) in^*a*^:					
	HPS/CTM; Roche^*b*^	CAP/CTM; Roche^*c*^	Versant; Siemens	Artus; Qiagen	RealTime; Abbott	Aptima; Hologic
LLOQ	25	15	15	35	12	10
LOD	9.3	15	15	21	12	5.1

a Data are from manufacturers' package inserts or manufacturers' websites. The target region for all tests is the 5′-UTR. The amplification method is PCR for all tests except Aptima, which uses TMA. The input volume is 0.5 ml for all tests except Artus, whose input volume is 1.0 ml.

b HPS/CTM, High Pure system/Cobas TaqMan version 2, considered the reference assay.

c CAP/CTM, Cobas AmpliPrep/Cobas TaqMan version 2.

The performances of some of these existing HCV quantitative assays have been compared side by side in various published studies (13, 23–26). However, the Aptima assay has not yet been evaluated in comparison to other assays. Thus, in the present study, we compared the performance of Aptima to that of HPS/CTM, the reference assay. The assay performances were compared using reference panels and clinical samples, and the parameters included agreement for HCV RNA detection and quantitation, sensitivity at low viral loads, linearity within the range of quantitation, and intra- and interassay reproducibility. The influence of genotypes was assessed for HCV genotypes 1, 2, and 3, the most prevalent genotypes worldwide (46%, 13%, and 22% prevalence, respectively) (27).

## MATERIALS AND METHODS

### Clinical samples.

Serum or plasma samples (from blood samples anticoagulated with EDTA) from HCV-positive patients (chronic cases) sent to Labor Prof. Gisela Enders & Kollegen, Stuttgart, Germany, were used in this study. Altogether, 267 samples (13 serum and 254 plasma samples) were collected, corresponding to the following inclusion/exclusion criteria. The inclusion criteria were patient age of ≥18 years of age, known HCV-positive status (based on commercially available nucleic acid amplification test [NAAT] or enzyme-linked immunosorbent assay [ELISA] results), leftover volume of at least 1.5 ml of the patient sample after routine testing, and sample storage at −70°C prior to side-by-side testing with Aptima and HPS/CTM. The exclusion criteria were patient age of <18 years, insufficient plasma or serum volume available for testing, invalid NAAT results, and unknown HCV infection status. The results for fresh and frozen samples are considered equivalent, according to the manufacturer, when the recommended collection and storage conditions are used.

### Ethical considerations.

The study was performed in accordance with the requirements of the ethical board of the local state chamber of physicians (Stuttgart, BW, Germany), and it was conducted in adherence to the Declaration of Helsinki. Only leftover samples from samples originally sent to our laboratory for routine HCV testing were used, and besides HCV RNA testing with HPS/CTM and Aptima in the context of this study, no other tests were performed. All samples were anonymized before beginning the study in that a unique identification (ID) number was assigned to each leftover sample and the study ID number did not contain any patient identifiers.

### Reference panels.

One commercially available panel and two external quality assessment (EQA) reference panels were used to test the assays for accuracy. The commercial panel was the Qnostics HCV evaluation panel QNCM14-038-HCV (Qnostics, Ltd., Scotland, United Kingdom), containing 8 panel members of genotypes 1b and 3a. The panel members were tested twice by Aptima and once by HPS/CTM, and the results were compared to the target values given by the manufacturer. The EQA panels included (i) the Instand HCV RNA panel, containing 8 members (all genotype 3) (Instand, Dusseldorf, Germany), and (ii) 4 HCV-positive panel members of the College of American Pathologists (CAP) HCV viral load survey panel HVL-A 2015 (CAP, Northfield, IL, USA). For the latter, no HCV genotype/subtype information was available. All EQA panel members were tested once in both assays, and the results were compared to the median target values generated by the EQA organizers.

### HCV RNA quantitation and genotyping assays.

All assays were performed according to the manufacturers' instructions.

### (i) Aptima.

The Aptima assay is a real-time TMA test for both detection and quantitation of HCV RNA of genotypes 1 through 6 in fresh and frozen human serum and plasma from HCV-infected individuals (22). The target sequence (HCV 5′-UTR) is first captured onto magnetic microparticles to allow separation from other plasma/serum components and then amplified using real-time TMA. The amplification products (amplicons) are detected and quantitated using fluorescently labeled probes. All steps (sample preparation, amplification, and detection) are performed in a fully automated manner on the Panther platform. The assay's LOD is 5.1 IU/ml or less across all genotypes, its LLOQ is 10 IU/ml, also demonstrated across all genotypes, and its dynamic range of quantitation is 10 to 10^8^ IU/ml. The test uses 0.5 ml of specimen input volume, and the time to first results for the assay is 2 h 41 min.

### (ii) HPS/CTM.

The HPS/CTM assay is a PCR-based real-time nucleic acid amplification test for the quantitation of HCV RNA genotypes 1 through 6 in human serum or plasma (Cobas TaqMan HCV test, version 2.0, 2013; Roche Molecular Systems). Specimen preparation, which consists of several steps of lysis and target RNA (5′-UTR) capture using glass particles, is performed manually using the generic High Pure system (Roche Diagnostics GmbH, Mannheim, Germany). Briefly, plasma was manually combined with a binding buffer and proteinase K and then added to a filter tube for several cycles of centrifugation and washing followed by elution. The amplification and detection steps are performed automatically by the Cobas TaqMan 48 analyzer. The assay's LOD for genotype 1 is 9.3 IU/ml in plasma and 8.8 IU/ml in serum. Its LLOQ is 25 IU/ml, with a dynamic range of 25 to 391 × 10^6^ IU/ml. The test uses 0.5 ml of specimen volume per test.

### (iii) LiPA.

The LiPA assay (Siemens Healthcare Diagnostics Ltd., Frimley, Camberley, United Kingdom) identifies HCV genotypes 1 through 6, including subtypes a and b of genotype 1. Biotinylated DNA PCR products, generated by reverse transcription (RT)-PCR amplification of the 5′-UTR and core regions of the HCV genome, are hybridized to immobilized specific probes (reverse hybridization). After hybridization, conjugate and substrate are added, resulting in a colored precipitate. This test does not quantitate HCV RNA. The LiPA assay was used to determine the HCV genotypes in clinical samples if the sample volume was sufficient.

### Preparation of samples to test variability, analytical sensitivity, and linearity.

To assess variability and sensitivity, 3 high-titer clinical samples containing HCV subtypes 1a, 1b, and 3a were chosen. For value assignment, the quantitative HPS/CTM results were used. The samples were then diluted with HCV-negative plasma to obtain the 3 target concentrations of 1,000, 100, and 25 IU/ml (3 log, 2 log, and 1.4 log IU/ml) based on the HPS/CTM results. The 25-IU/ml concentration was chosen because it is the minimum required LLOQ for an assay (5). Ten replicates of each dilution were tested within the same run over 3 consecutive days with each assay (total of 30 data points per dilution). Intra-assay variability was based on 10 replicates in a single day, while total variability was based on the cumulative results for 3 days (30 replicates).

The linearity and the influence of HCV genotype were assessed using 4 clinical samples, containing HCV subtypes 1a, 1b, 2b, and 3a (the most represented worldwide [27]), which were diluted to obtain 7 target concentrations (6, 5, 4, 3, 2, 1.7, and 1.4 log IU/ml). Five replicates of each dilution level were tested side by side in each assay. Serial dilutions and replicates were prepared using HCV-negative EDTA-plasma as the diluent (SeraCare, Life Sciences, Milford, MA) (the HCV-negative status was confirmed by retesting in HPS/CTM and Aptima assays). The dilutions were aliquoted according to the test protocol, and the aliquots were treated identically, with exactly the same number of freeze/thaw cycles.

### Data analyses.

Clinical samples were excluded from the analyses if they yielded an invalid result in one or both assays. The agreement between assay results for HCV negativity and positivity was assessed by tabulation of paired data and by calculating the degree of agreement (kappa) and associated 95% confidence interval (CI) for each pairwise comparison. For clinical samples quantitated by both assays, agreement between the assays was assessed using (i) Deming regression analysis with calculation of the correlation coefficient (using Pearson's correlation) and (ii) Bland-Altman analysis, which plots the difference in measurements between two assays against the average of the two assays. Differences were tested using paired *t* tests for each pairwise comparison. Intra-assay reproducibility was assessed by calculating the coefficients of variation (CoVs) for the viral load values obtained in each assay for the 30 replicates tested. Interassay reproducibility was assessed by calculating the proportions of samples in which HCV RNA was detected and quantifiable among the 30 replicates tested. All analyses were performed using GraphPad Prism 6 (GraphPad Software, Inc., San Diego, CA).

## RESULTS

### Comparison of Aptima and HPS/CTM using reference panels.

Comparison of the Aptima and HPS/CTM results in the 3 HCV reference panels revealed high agreement between the assays, with differences between the assay results of less than 0.5 log (except for one data point in the CAP panel where the difference was 0.56 log) (Table 2). The HPS/CTM results more closely matched the panel target values, with differences of less than 0.5 log, while Aptima measured one sample in the Instand panel (sample number 379013) as 0.56 log lower than the assigned value (Table 2).

**TABLE 2:** Measurements of HCV RNA in the Instand, Qnostics, and CAP reference panels by Aptima and HPS/CTM assays

Panel	Panel member	Target concn (log_10_ IU/ml)	No. of replicates	Value (log_10_ IU/ml) for:				
				Mean concn in Aptima	Difference from target	Mean concn in HPS/CTM	Difference from target	Difference between assays (Aptima − HPS/CTM)
Instand	362085	4.06	1	3.74	0.32	3.98	0.08	−0.24
	362086	3.17	1	2.72	0.45	3.04	0.13	−0.32
	362087	4.56	1	4.29	0.27	4.44	0.12	−0.15
	362088	3.65	1	3.28	0.37	3.52	0.13	−0.24
	379013	3.19	1	2.63	0.56	3.08	0.11	−0.45
	379014	1.48	1	1.00^*a*^		1.40^*b*^		−0.40
	379015	1.80	1	1.54	0.26	1.60	0.20	−0.06
	379016	2.76	1	2.37	0.39	2.59	0.17	−0.22
Qnostics	14383A18	1.8	2^*c*^	1.45	−0.35	1.89	0.09	−0.44
	1438NOO	0	2	0	0	0	0	0
	14383A28	2.8	2	2.42	−0.38	2.76	−0.04	−0.34
	14383A28	2.8	2	2.45	−0.35	2.69	−0.11	−0.24
	14383A38	3.8	2	3.37	−0.43	3.66	−0.14	−0.29
	14381B23	2.3	2	2.27	−0.03	2.54	0.24	−0.27
	14381B33	3.3	2	3.32	0.02	3.46	0.16	−0.14
	14381B43	4.3	2	4.25	−0.05	4.53	0.23	−0.28
CAP^*d*^	HCV201.2015	2.70	1	2.90	0.2	2.53	−0.17	0.37
	HCV202.2015	3.72	1	4.05	0.33	3.49	−0.23	0.56
	HCV203.2015	5.09	1	5.25	0.16	4.89	−0.20	0.36
	HCV205.2015	3.73	1	3.72	−0.01	3.51	−0.22	0.21

a Aptima HCV was assigned a value of 1.00 when RNA was detected but not quantified.

b HPS/CTM HCV was assigned a value of 1.40 when RNA was detected but not quantified.

c Only 1 replicate was tested with HPS/CTM.

d The CAP results for HPS/CTM are historical results.

### Comparison of Aptima and HPS/CTM using patient serum and plasma samples.

Altogether, 267 samples (13 serum and 254 EDTA plasma samples) collected between 2014 and 2015 from HCV-positive patients were tested in parallel using the Aptima and HPS/CTM. One sample with an invalid Aptima result was excluded from the analysis because repeated testing was not possible. Valid results were obtained with both tests for 266 samples. Genotype (GT) information was available from 164/266 samples, with the following distribution: GT 1, 122 samples (74.4%); GT 2, 10 samples (6.1%); GT 3, 27 samples (16.5%); and GT 4, 5 samples (3%). No genotype information was available from the remaining 102 samples, and the sample volumes were not sufficient for genotyping.

The overall concordance between the assays for discrimination between negative and positive samples (grouping samples in which HCV RNA was <LLOQ but detected and samples in which it was quantitated) was 92.1% (245/266) (kappa = 0.80; 95% CI, 0.720 to 0.881) (Table 3).

**TABLE 3:** Agreement between Aptima and HPS/CTM for the detection and quantitation of HCV RNA in patients' plasma samples

Outcome in HPS/CTM	No. of samples with outcome using Aptima^*a*^			Total no.
	Negative	<LLOQ (10 IU/ml)	Quantitated	
Negative	61	15	5	81
<LLOQ (25 IU ml)	0	2	17	19
Quantitated	1	0	165	166
Total	62	17	187	266

a Positive samples include samples with <LLOQ and quantitated samples.

Twenty samples were detected as positive with Aptima, 5 at quantifiable levels (4 GT 1a and 1 GT 1b) and 15 at levels below the LLOQ (5 GT 1a, 8 GT 1b, and 1 GT 2b, with no GT available for one sample), but were all negative in HPS/CTM. One sample was detected as positive in HPS/CTM (at a low but quantifiable level, with no GT available) but was negative in Aptima. Two samples were detected with both assays at levels below their LLOQs (1 GT 1a and 1 with the GT not available). Seventeen (7 GT 1a, 6 GT 1b, and 4 GT not available) samples were quantifiable by Aptima and positive but below its LLOQ by HPS/CTM. Further analysis of these discrepant results with a third system was not possible due to insufficient sample volume.

**FIG 1:**
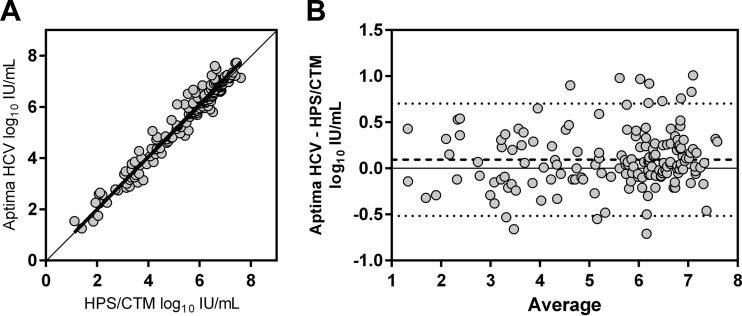
Correlation between assays for patient samples. (A) Correlation analysis with samples quantified by both assays. *Y* = 1.026*x* − 0.04816; Pearson *r* = 0.9798; *P* < 0.0001. (B) Bland-Altman analysis of agreement between Aptima and HPS/CTM. The bold dashed line indicates the mean difference in assay values (Aptima − HPS/CTM). The two dotted lines indicate the 95% CI of the assays' agreement.

For the 165/266 (62.0%) samples quantitated by both assays, the viral loads ranged from 1.11 to 7.73 log IU/ml in Aptima and from 1.40 to 7.60 log IU/ml in HPS/CTM. Deming regression analysis of the 165 paired results showed a good correlation between the assays' viral load values (Pearson's *R* = 0.98; *P* < 0.0001) (Fig. 1A). Bland-Altman analysis showed that the difference between the mean results of the assays was small (0.094 log) and that 92.1% of the sample results lay within the 95% CI of the agreement (Fig. 1B).

### Linearity by genotype.

**FIG 2:**
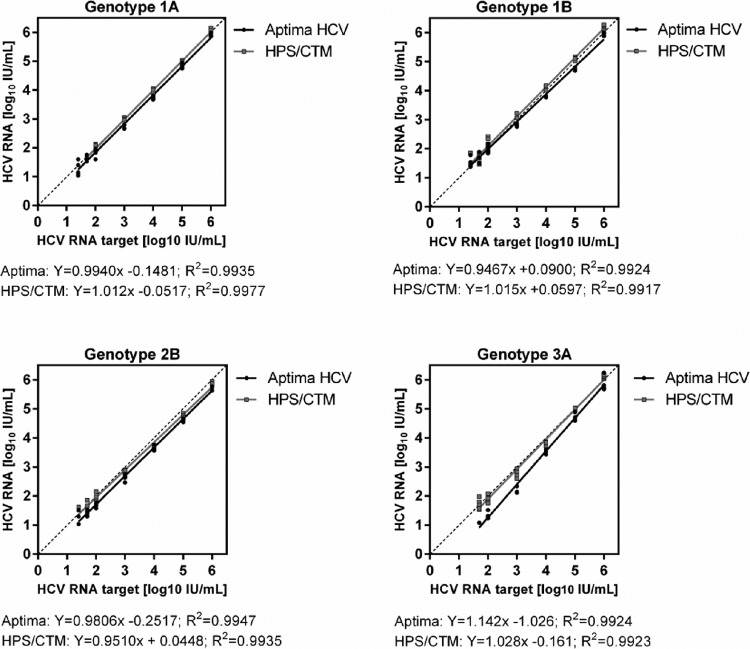
Linearity and influence of HCV genotype. Linearity was assessed using 4 clinical samples containing HCV subtypes 1a, 1b, 2b, and 3a, with seven dilution levels and target concentrations (in IU/ml) of 6 log, 5 log, 4 log, 3 log, 2 log, 1.7 log, and 1.4 log. Five replicates of each dilution level were tested side by side in each assay.

The linearity of quantitation from 1.4 log to 6 log was excellent for both assays for all genotypes tested (1a, 1b, 2b, and 3a), as demonstrated by correlation factors (*R*^2^) ranging from 0.9917 to 0.9977 (Fig. 2).

### Total and intra-assay variability.

As expected, the variability of the results in both assays increased as the HCV RNA concentration decreased (Table 4). For genotypes 1a and 3a, the levels of total variability were similar for both assays, with the percent coefficient of variation (%CoV) values ranging from 2.55% to 4.16% for the concentration of 1,000 IU/ml target, 3.92% to 7.02% for 100 IU/ml, and 10.01% to 12.94% for 25 IU/ml (Table 4). For genotype 1b, the levels of variability were similar for the two assays at the 1,000-IU/ml target concentration (4.14% versus 4.41%), higher for the Aptima at 100 IU/ml (11.59% versus 7.82%), and not comparable for 25 IU/ml due to too few replicates being quantitated at that concentration (Table 4). The intra-assay variability on each day (10 replicates per assay) for Aptima HCV across genotypes ranged from 1.31% to 5.06% at 1,000 IU/ml, 4.31% to 15.00% at 100 IU/ml, and 6.96% to 15.20% at 25 IU/ml (data not shown). For HPS/CTM, the values were 1.11% to 3.91% at 1,000 IU/ml, 2.39% to 8.62% at 100 IU/ml, and 5.37% to 8.52% at 25 IU/ml (only GT 3a was included at 25 IU/ml due to the lack of replicates with quantitative values each day for the other genotypes).

**TABLE 4:** Variability of Aptima HCV and HPS/CTM results using clinical samples of three different genotypes at three different dilutions^*a*^

Genotype	Assay	Concn (log IU/ml)	No. of replicates	Mean	SD	Median	25th–75th percentile	Minimum–maximum	%CoV
1a	Aptima	3.0	30	2.82	0.10	2.84	2.73–2.88	2.60—3.00	3.71
		2.0	30	1.90	0.13	1.92	1.81–1.98	1.57–2.16	7.02
		1.4	22	1.27	0.15	1.26	1.14–1.40	1.08–1.63	11.93
	HPS/CTM	3.0	30	2.79	0.07	2.79	2.74–2.84	2.67–2.99	2.55
		2.0	30	1.95	0.12	1.96	1.84–2.04	1.70–2.15	6.35
		1.4	5	1.57	0.20	1.46	1.42–1.78	1.40–1.87	12.94
1b	Aptima	3.0	30	2.29	0.09	2.29	2.23–2.37	2.05–2.47	4.14
		2.0	27	1.30	0.15	1.28	1.23–1.40	1.00–1.58	11.59
		1.4	4	1.26	0.25	1.22	1.04–1.51	1.04–1.54	20.30
	HPS/CTM	3.0	30	2.56	0.11	2.57	2.50–2.66	2.38–2.77	4.41
		2.0	22	1.59	0.12	1.58	1.48–1.68	1.43–1.84	7.82
		1.4	ND^*b*^	ND	ND	ND	ND	ND	ND
3a	Aptima	3.0	30	2.86	0.10	2.84	2.79–2.93	2.61–3.04	3.69
		2.0	30	1.95	0.12	1.95	1.84–2.04	1.72–2.19	6.25
		1.4	28	1.32	0.13	1.30	1.23–1.41	1.04–1.60	10.01
	HPS/CTM	3.0	30	3.08	0.13	3.13	2.95–3.19	2.83–3.25	4.16
		2.0	30	2.32	0.09	2.30	2.24–2.40	2.15–2.49	3.92
		1.4	18	1.71	0.18	1.67	1.55–1.88	1.46–2.10	10.75

a Samples were diluted to 1,000, 100, and 25 IU/ml (3.0 log, 2.0 log, and 1.4 log IU/ml, respectively).

b ND, no data.

### Analytical sensitivity.

The overall detection rates for the assays were similar, ranging from 100% for replicates at 1,000 and 100 IU/ml HCV RNA to 98.9% (Aptima) and 96.7% (HPS/CTM) for replicates at 25 IU/ml (Table 5). Although both assays quantitated 100% of the replicates at 1,000 IU/ml, Aptima was capable of quantitating more replicates than HPS/CTM at lower target concentrations (96.7% versus 90.0% at 100 IU/ml and 60.0% versus 40.0% at 25 IU/ml, respectively) (Table 5). While both assays detected all genotypes similarly well at all concentrations tested (1,000, 100, and 25 IU/ml), quantitation of genotype 1b was limited for both assays at the nominal concentration of 25 IU/ml, with HPS/CTM unable to quantify any of the 30 replicates and Aptima quantifying 4 out of 30 (Table 5).

**TABLE 5:** Analytical sensitivities of Aptima HCV and HPS/CTM tests using clinical samples of three different genotypes at different dilutions

Assay	Concn (IU/ml) of target	Results (no. of samples in which HCV RNA was detected/no. in which it was quantitated) for genotype:			Overall rate (%) of:	
		1a	1b	3a	Detection	Quantitation
Aptima	1,000	30/30	30/30	30/30	100	100
	100	30/30	30/27	30/30	100	96.7
	25	30/22	29/4	30/28	98.9	60
HPS/CTM	1,000	30/30	30/30	30/30	100	100
	100	30/30	30/21	30/30	100	90
	25	30/18	27/0	30/18	96.7	40

## DISCUSSION

The guidelines for monitoring HCV-positive patients during treatment recommend the use of a highly sensitive real-time quantitative HCV RNA assay with an LOD of ≤15 IU/ml and an LLOQ of ≤25 IU/ml (5). Although several commercially available assays have been cleared for the detection and quantitation of HCV RNA in patients' plasma or serum samples, their performances vary widely, with LODs from 5.1 to 21 and LLOQs from 10 to 35 IU/ml (Table 1). Of all currently available assays, the newly approved Aptima assay has the lowest LOD and LLOQ (5.1 and 10 IU/ml, respectively) and has been approved with claims for both diagnosis and monitoring in Europe (CE-approved IVD; Aptima is not currently U.S. FDA approved for clinical monitoring). Here, we compare for the first time the performance of the Aptima assay to that of HPS/CTM, which is considered the reference assay for HCV RNA quantitation in patients' plasma and serum samples during antiviral treatment. We found the Aptima's performance to be similar to that of HPS/CTM in many aspects, as the two assays (i) had good overall agreement of results in 3 reference panels (differences of <0.5 log), (ii) good agreement for detection/nondetection of HCV RNA in clinical samples (92.1%), (iii) good agreement for quantitative values in samples quantitated by both assays (average difference of 0.094 log), (iv) similarly excellent linearity within the range of quantitation, (v) similar levels of intra-assay reproducibility, and (vi) similar rates of detection and quantitation of replicates at 1,000 and 100 IU/ml. However, the analytical performance of Aptima was superior to that of HPS/CTM in two instances: (i) Aptima detected or quantitated 20 clinical samples that were missed (negative) by HPS/CTM (while Aptima only missed 1 sample that was quantitated by HPS/CTM) and (ii) Aptima quantitated 50% more replicates than HPS/CTM did at the 25-IU/ml concentration of the target (in the interassay reproducibility experiment). Further experiments with larger sample populations are necessary to examine the significance of the improved sensitivity of Aptima in patient diagnosis and to investigate its utility in clinical monitoring.

Recent studies have compared HPS/CTM to other commercially available assays and shown that at 100 and 25 IU/ml, the intra-assay CoVs are higher than those of either the Abbott RealTime HCV test or the Roche Cobas AmpliPrep/Cobas TaqMan HCV test (13). Interestingly, in our study, the precision of HPS/CTM was higher than previously shown in these studies. Our data indicate that Aptima has precision (10% to 20% at 25 IU/ml) that is comparable to that of HPS/CTM at these low levels (10.75% to 20.3% at 25 IU/ml) for genotypes 1a and 3a but superior for genotype 1b (20.3% CoV), since HPS/CTM failed to quantitate these samples at 25 IU/ml. This indicates that Aptima may have higher precision than RealTime HCV (16% to 35% at 25 IU/ml) and Cobas TaqMan HCV test (28% to 55% at 25 IU/ml). Direct comparison will be necessary to verify these findings, as the study designs were different (single versus multicenter and different genotypes) and the numbers of replicates not identical (10 versus 30). High precision at 25 IU/ml is of major importance to properly assess SVR and should be taken into consideration when switching assays during therapy.

These findings suggest that Aptima has a greater analytical sensitivity (LOD). Thus, although the HPS/CTM assay can be considered a reference test for quantitating HCV RNA in patients' clinical samples during DAA treatment, we question whether it truly meets the requirement of the recent guidelines (LLOQ of ≤25 IU/ml) (5) for use in monitoring treatment. With an LLOQ well below 25 IU/ml (10 IU/ml), the Aptima assay offers a more adequate LLOQ. Some studies following longitudinal cohorts of patients have observed that monitoring with a more sensitive assay may be more predictive of long-term clinical outcomes and relapse (28, 29).

Sensitivity, precision, and accuracy are important characteristics to consider when choosing an assay to monitor patients during treatment, since only a single measurement is used to predict whether treatment is effective (i.e., the patient has achieved SVR). We show herein that Aptima is a highly sensitive, accurate, and reproducible assay, with performance equal or superior to that of HPS/CTM. Thus, Aptima can be recommended for the quantitation of HCV RNA in clinical samples of HCV-infected patients to monitor treatment efficacy in regions where it is currently approved for that purpose.
